# Metagenomic Insights into Rhizospheric Microbiome Profiling in Lentil Cultivars Unveils Differential Microbial Nitrogen and Phosphorus Metabolism under Rice-Fallow Ecology

**DOI:** 10.3390/ijms21238895

**Published:** 2020-11-24

**Authors:** Krishnendu Pramanik, Arpita Das, Joydeep Banerjee, Anupam Das, Shayree Chatterjee, Rishu Sharma, Shiv Kumar, Sanjeev Gupta

**Affiliations:** 1 Bidhan Chandra Krishi Viswavidyalaya, Mohanpur, Nadia, West Bengal 741252, India; kpramanik7@gmail.com (K.P.); arpitacoh@gmail.com (A.D.); cshayree@gmail.com (S.C.); rrishu.sharma90@gmail.com (R.S.); 2 Agricultural and Food Engineering Department, Indian Institute of Technology Kharagpur, West Bengal 721302, India; jbanerjeebiotech@gmail.com; 3 Department of Soil Science and Agricultural Chemistry, Bihar Agricultural University, Sabour, Bhagalpur, Bihar 813210, India; anusoil22@gmail.com; 4 International Centre for Agricultural Research in the Dry Areas (ICARDA), Rabat-Institutes, Rabat B.P. 6299, Morocco; 5 All India Coordinated Research Project (AICRP) on MULLaRP, ICAR—Indian Institute of Pulses Research, Kanpur, Uttar Pradesh 208024, India

**Keywords:** metagenomics, microbiome diversity, phosphorus metabolism, rice-fallow, lentil

## Abstract

The plant rhizosphere interfaces an array of microbiomes related to plant growth and development. Cultivar-specific soil microbial communities with respect to their taxonomic structure and specific function have not been investigated explicitly in improving the adaptation of lentil cultivars under rice-fallow ecology. The present study was carried out to decipher the rhizosphere microbiome assembly of two lentil cultivars under rice-fallow ecology for discerning the diversity of microbial communities and for predicting the function of microbiome genes related to nitrogen (N) and phosphorus (P) cycling processes deploying high-throughput whole (meta) genome sequencing. The metagenome profile of two cultivars detected variable microbiome composition with discrete metabolic activity. Cyanobacteria, Bacteroidetes, Proteobacteria, Gemmatimonadetes, and Thaumarchaeota were abundant phyla in the “Farmer-2” rhizosphere, whereas Actinobacteria, Acidobacteria, Firmicutes, Planctomycetes, Chloroflexi, and some incompletely described procaryotes of the “Candidatus” category were found to be robustly enriched the rhizosphere of “Moitree”. Functional prediction profiles of the microbial metagenomes between two cultivars revealed mostly house keeping genes with general metabolism. Additionally, the rhizosphere of “Moitree” had a high abundance of genes related to denitrification processes. Significant difference was observed regarding P cycling genes between the cultivars. “Moitree” with a profuse root system exhibited better N fixation and translocation ability due to a good “foraging strategy” for improving acquisition of native P under the nutrient depleted rice-fallow ecology. However, “Farmer-2” revealed a better “mining strategy” for enhancing P solubilization and further transportation to sinks. This study warrants comprehensive research for explaining the role of microbiome diversity and cultivar–microbe interactions towards stimulating microbiome-derived soil reactions regarding nutrient availability under rice-fallow ecology.

## 1. Introduction

Plant roots interface diverse groups of microbiotas including bacteria, fungi, algae, viruses, and archaea in soil [1,2], which are considered the host’s second genome [3]. The symbiotic association between plants and microbes leads to better adaptation in relation to plant growth, nutrient cycling, pathogen resistance, and stress tolerance, and in exchange, plants provide 20% of their fixed carbon (C) and 15% of nitrogen (N) to microbiomes for their growth and proliferation [4,5,6]. Specific strains of fungi and bacteria incline towards a particular crop cultivar through a network of plant–microbe interactions mediated by plant molecular signaling, differences in plant root morphology, quantity of rhizo-deposits, and root exudates [7,8]. The dynamic rhizospheres’ microbiome composition further alters microbe-driven soil functions as well as the potentiality of harnessing crop growth and productivity by stimulating beneficial plant–microbe interaction [9,10,11].

Lentil (*Lens culinaris* Medikus subsp. *culinaris*) is a globally important cool-season food legume which plays a major role in household nutritional security in developing countries. This protein-rich crop is an ideal candidate for rice-fallow areas aimed at its intensification to meet the protein demand of vegetarian society. In South Asia, about 14 million ha area remain fallow after the rice harvest covering India, Bangladesh, Nepal, and Pakistan, with India alone accounting for 79% of area [12]. The availabilities of N and phosphorus (P) are the key determining factors for maintaining soil health and crop productivity [13]. Rice-fallow areas are characterized by poor soil N and inorganic P status as well as soil organic C [14]. Soils of rice-fallow areas are different from the soils of other lentil growing areas due to alterations from the anaerobic condition during rice growing season to the aerobic condition which strikingly alters the microbial population dynamics, leading to a reduction in biological nitrogen fixation (BNF) and P availability in the succeeding lentil crop [15,16].

Lentil plants maintain symbiotic association with diverse microbial populations for improving soil health and crop production through BNF [17]. The host genotype, *Rhizobium* strain, and cultivar-specific interactions influence BNF in lentil [18]. Bacteria play the major role in BNF processes and exhibit considerable diversity in the rhizosphere. BNF is primarily a reduction process mediated by the nitrogenase enzyme [19] encoded by a core of *nif* genes including *nifH*, *K*, *B*, *E*, *N*, *Z*, and *X* underlying nitrogenase synthesis and catalysis [20,21]. Moreover, lentil also depends on *Rhizobium* symbionts for other N cycling process like N mineralization, immobilization, and various oxidation–reduction reactions for transforming different forms of N mediated by microbiome communities [22,23]. Lentil craves adequate P supply for satisfactory nodule production and subsequent N fixation. Plant and microbes adopted three important strategies for improving production under P limiting soils: firstly, cultivars with prolific root systems develop a root “foraging strategy” for improving acquisition of soil P [24]; secondly, the soil P “mining strategy” augments desorption, solubilization, or mineralization of P from either sparingly available pools or from resistant organic pools [25,26]; and thirdly, “internal P-utilization efficiency” is improved [26,27]. Additionally, some specific soil microbiomes indirectly influence the mineralization of different organic forms of P [28] by intensifying the P recycling process for transformation of soil P_i_ (inorganic) and P_o_ (organic) into available forms through overexpression of phosphatase. Therefore, microorganisms impart a pivotal role in maintaining P_o_ and P_i_ pools in the soil [29].

The abundance and diversity of a rhizospheric microbiome among the plant species are highly influenced by the plant genetic traits [30,31] as well by the soil physical and chemical properties [32,33]. Earlier studies with a cultivar response towards the microbial community structure and diversity in peanut [6] and maize [34] revealed that cultivars differing in N use efficiency or BNF had a differential microbiome community with diverse enzymatic activity. In sweet potato, the composition of phosphate-mineralizing bacterial community exhibited significant diversity depending on genotypic differences [35]. Similarly, different soybean genotypes recruited diverse *Rhizobium* colonies and mildly tuned rhizosphere microbiome assembly [11].

Recently, Next Generation Sequencing (NGS)-based OMICS technologies provided better insight into the crucial roles of rhizosphere microbiomes towards soil and plant genetic traits under varying conditions [33,36,37]. Comprehensive research has not yet been carried out in lentil towards deciphering the genotype-dependent microbiome diversity along with the studies on additional changes in the soil geochemical reactions towards availability of important nutrients for plant growth. The intrinsic adaptability of lentil cultivar under a rice-fallow situation is also unknown. Keeping these facts in view, a whole metagenome sequencing approach was employed to decipher the diversity and abundance of a microbial population associated with two differentially performing lentil cultivars under rice-fallow ecology. In addition to that, the predicted function of the microbiome genes towards N and P cycling processes in two lentil cultivars was also investigated.

## 2. Results

### 2.1. Plant Attributes of the Test Cultivars

**Figure 1:**
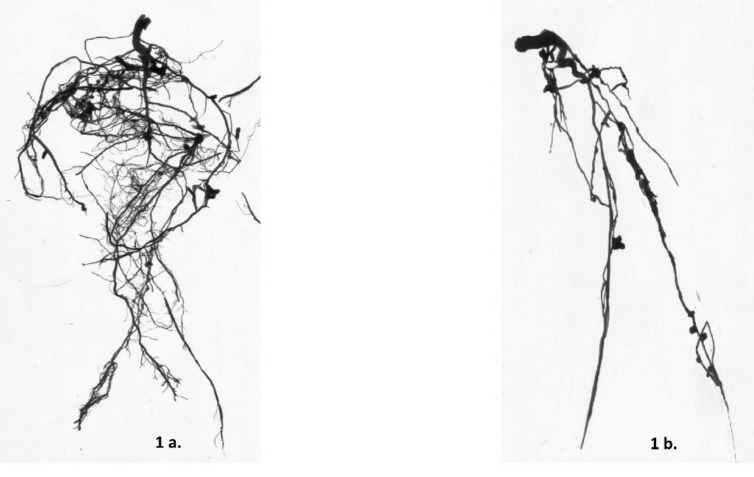
Root scan of “Moitree” (**a**) and “Farmer-2” (**b**) under rice-fallow ecology in WINRHIZO software package.

Both the lentil cultivars responded in significantly different (*p* = 0.034) manner under rice-fallow ecology. The varied performance of lentil cultivars under rice-fallow ecology was attributed to their divergent grain nutrient content, shoot–root ratio, and root characteristics. Grain nutrient content of lentil cultivars varied significantly (Table 1). Grain N content was higher in “Moitree” (4.47%) than in “Farmer-2” (3.64%), whereas P content was higher in “Farmer-2” (2.74%) compared to “Moitree” (2.61%). Root N and P contents were higher in “Farmer-2” (N 0.98%, P 1.72%) in comparison to “Moitree” (N 0.65%, P 0.96%). Both cultivars, Moitree (8.41) and Farmer-2 (4.63), also differed significantly in shoot–root ratio considering their dry weight of shoot and root. This could be attributed to the better nutrient translocation efficiency of “Moitree”. Moreover, enzymatic activity in relation to P nutrition also differed as phytase and acid phosphatase activities were higher in “Farmer-2” in comparison to ‘’Moitree’’. However, enzymatic activities related to N metabolism viz. allantoin and leghaemoglobin (LB) were better in “Moitree” than in “Farmer-2”. The nodule number was higher in “Moitree” as compared to “Farmer-2”. Results showed that “Moitree” had profuse root growth under rice-fallow condition that was attributed to higher average tap root length (38.97 cm), root surface area (29.7 cm^2^), and root diameter (0.7 mm) than “Farmer-2” (Table 1 and Figure 1).

### 2.2. Enzymatic Activity in Soil

Phosphatase activity in soil indicates the ability of a plant species and/or their associated microbial community to utilize native inorganic P for plant nutrition; however, phytase activity designates P utilization from native organic P. It was found that “Farmer-2” attributed higher acid phosphatase (42.9 μg *p*-nitrophenol/g soil/hr), alkaline phosphatase (36.9 μg *p*-nitrophenol/g soil/hr), and phytase (2.32 mg P/g soil/hr) activity in soil as compared to “Moitree” (Table 2). Data indicated that “Farmer-2” facilitated 25.6, 24.7, and 7.8% more acid phosphatase, alkaline phosphatase, and phytase activity, respectively, over “Moitree”.

### 2.3. Comparison of the Rhizospheric Microbiome Profiles between Two Lentil Cultivars

**Figure 2:**
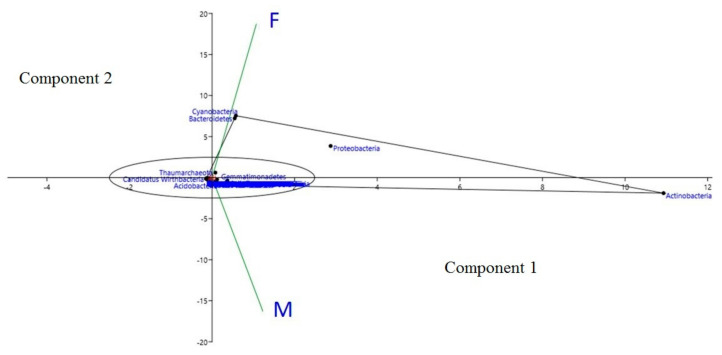
Principal component analysis (PCA) of rhizospheric microbes associated with lentil cultivars “Moitree” (M) and “Farmer-2” (F) under rice-fallow ecology based on shared common taxa profiles at phylum abundance variance.

**Figure 3:**
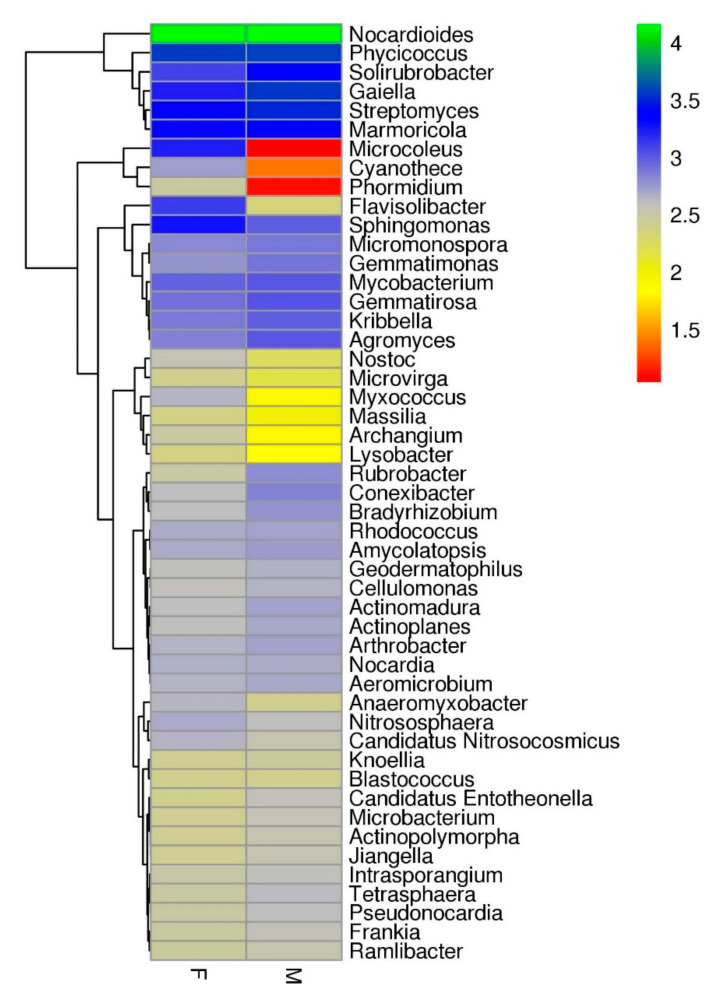
Relative abundance of the top 50 common bacterial genera within microbial communities sampled from lentil cultivars Moitree (M) and Farmer-2 (F) using R package Pheatmap.

Analysis of the taxonomic community exhibited that the environments were dominated by bacteria (97% in “Farmer-2” and 98% in “Moitree”). The remaining sequences matched Archaea (2% for both “Farmer-2” and “Moitree”) and Eukaryota (0.02% in “Farmer-2” and 0.2% in “Moitree”) or were uncategorised (0.006% in “Farmer-2” and 0.06% in “Moitree”). Enrichment of the phyla Actinobacteria and Proteobacteria was found at maximum in the samples of “Farmer-2” and “Moitree”, respectively. A large proportion of the sequences were unclassified at the phylum level (approximately 22.4% of sequences within “Farmer-2” and 23.77% for “Moitree”). The most abundant top 50 bacterial taxa within microbial communities between “Farmer-2” and “Moitree” were identified, and a comparative heat tree was generated (Supplementary Figure S1). A principal component analysis (PCA) plot with both phylum read and abundance variance highlighted the fact that Cyanobacteria, Bacteroidetes Proteobacteria, Gemmatimonadetes, and Thaumarchaeota were abundant in “Farmer-2” whereas Actinobacteria, Acidobacteria, Firmicutes, Planctomycetes, Chloroflexi, and unclassified procaryotes in the category of Candidatus were most prevalent in “Moitree” (Figure 2 and Supplementary Figure S2). A genera level heat tree also predicted that *Nocardioides* was the most abundant genus in both cultivars (7.49% in “Farmer-2” and 7.04% in “Moitree”) (Figure 3).

**Figure 4:**
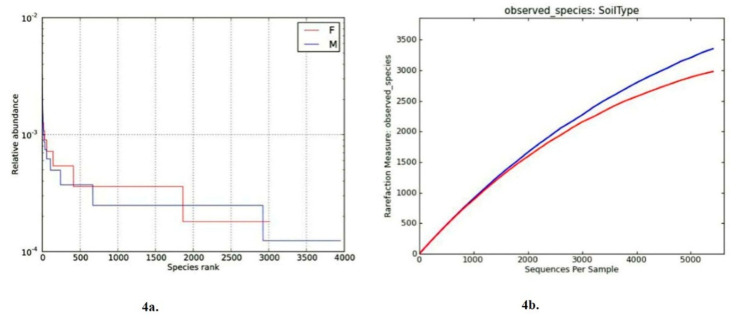
Microbial diversity in “Moitree” and “Farmer-2” cultivars: (**a**) rank–abundance curves for lentil cultivars Moitree (M) and Farmer-2 (F) resulting from Next Generation Sequencing (NGS) reads from the V5–V6 region of 16S rRNA genes from Bacteria and Archaea, in which Operational taxonomic units (OTUs) are based on 97% sequence similarities, and (**b**) rarefaction curves of OTUs (by Chao1 estimates) of lentil cultivars Moitree (M) and Farmer-2 (F) under rice-fallow ecology.

Strikingly, among the most abundant bacterial genera, *Paenibacillus* was found only in “Farmer-2”. Moreover, most predominant genera exhibited differences between “Farmer-2” and “Moitree”. Specifically, the relative abundances of *Sphingopyxis* and *Novosphingobium* (belonging to Sphingomonadaceae), *Lysobacter* (belonging to Xanthomonadaceae), and *Luteimonas* (belonging to Xanthomonadaceae) were more in “Farmer-2” than in “Moitree”, but the relative abundances of *Streptomyces* (belonging to Streptomycetaceae) and *Pseudomonas* (belonging to Pseudomonadaceae) were higher in “Moitree”. The estimated bacterial diversity did not vary considerably between two lentil cultivars (Table 3). No statistically significant differences (*p* > 0.05) in alpha diversity were found for Shannon index among the observed species and Chao 1 under different cultivars, though microbiome composition of soils associated with “Moitree” reflected relatively high diversity in comparison to “Farmer-2”. Species richness can be viewed as the number of different species on the chart, and species evenness was derived from the slope of the line that fits the “Rank Abundance Plot”. A steep gradient in rank abundance curve exhibited by “Moitree” indicated low evenness, whereas in “Farmer-2”, it reflected that a few phylotypes comprised a major proportion of the rhizosphere microbiome where more communities were seen in “Moitree” (Figure 4a). Rarefaction curves of the OTUs (operational taxonomic units) in roots of “Farmer-2” and “Moitree” are depicted in Figure 4b. A similar steep slope in the Rarefaction curve also indicated that a large fraction of the species diversity remains to be discovered, and this finding warrants further sequencing of the samples.

### 2.4. Comparison of the Functional Profiles for the Microbial Metagenomes

**Figure 5:**
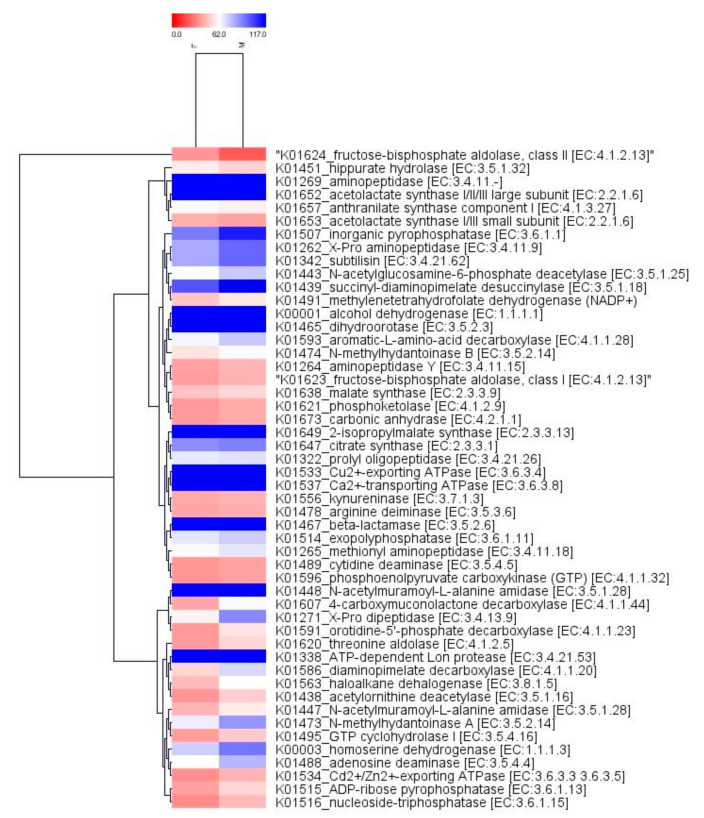
Heat tree of the top 50 common KEGG (Kyoto Encyclopedia of Genes and Genomes) KO groups in lentil cultivars Moitree (M) and Farmer-2 (F) using the MeV tool.

**Figure 6:**
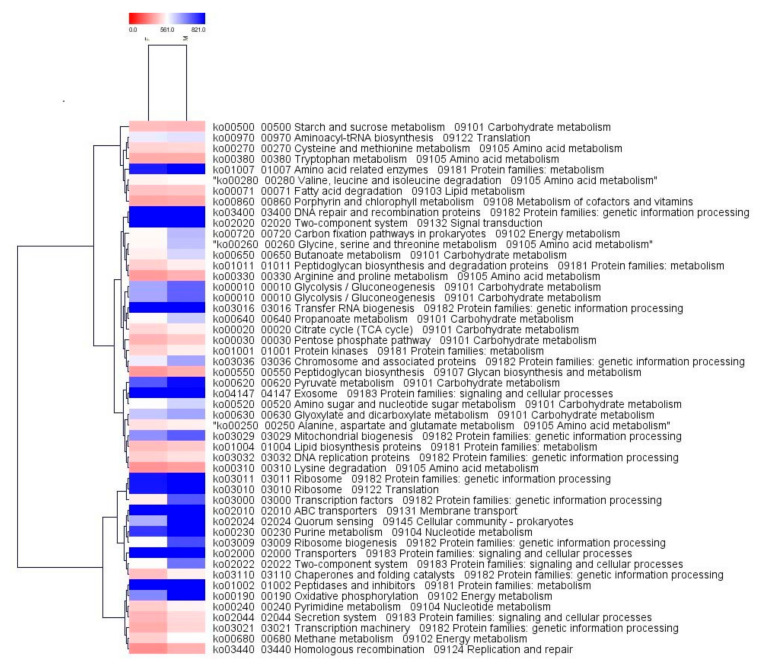
Heat tree of the top 50 common KEGG (Kyoto Encyclopedia of Genes and Genomes) pathways using the MeV tool for lentil cultivars Moitree (M) and Farmer-2 (F).

The maximum numbers of hit were observed against Gene Ontology (GO), followed by the Kyoto Encyclopedia of Genes and Genomes (KEGG) and protein families (Pfam) databases in both samples. In the case of “Farmer-2”, 102,466 genes from the total of 186,216 genes exhibited hit at least in one database, whereas in “Moitree”, 122,803 genes were identified showing hit at least in one database out of 206,155 genes. The GO project provides controlled vocabularies of defined terms representing gene product properties. GO functional analysis assigned a total of 147,839 genes in “Farmer-2” and 165,424 genes in “Moitree” while KEGG functional analysis revealed that 120,946 genes have been assigned with 4300 KEGG classes in “Farmer-2” and that 140,995 genes have been assigned with 4336 KEGG classes in “Moitree”. Relative abundances of the top 50 KEGG Orthology (KO) groups which were common in both samples were identified, and a comparative heat tree was generated (Figure 5). The maximum assigned KO belonged to the metabolism category followed by environmental and information processing and genetic information processing in both samples, and these were relatively higher in “Moitree” than in “Farmer-2”. KEGG KAAS (KEGG Automatic Annotation Server) was performed individually for both the samples, followed by identifying common KEGG pathways, and a heat tree was generated for the top 50 (Figure 6).

COG (Clusters of Orthologous Groups of proteins) functional analysis revealed that 96,574 genes have been assigned with COG functions in “Farmer-2” where 11,364 genes fall in the category of general function prediction and 10,253 genes belong to the amino acid transport and metabolism category. However, in “Moitree”, COG functional analysis revealed that 116,357 genes have been assigned with COG functions, where maximum genes (13,873) fall in the category of general function prediction and 12,868 genes belong to amino acid transport and metabolism. Pfam functional annotation was used to assess protein families and domain. Pfam functional analysis showed that 109,486 gene sequences have been assigned, with a total of 3889 Pfam domains in “Farmer-2”, whereas in ‘Moitree’, 124,850 gene sequences have been assigned, with a total of 3813 Pfam domains. FIGfams (Fellowship for the Interpretation of Genomes protein families) are sets of protein sequences that are similar along their full length. Thus, all the proteins within a single FIGfam are believed to implement the same function. In other words, FIGfam is a set of functional homologs. FIGfam functional analysis revealed that 61,058 genes have been assigned, with a total of 6933 FIG (Fellowship for the Interpretation of Genomes) classes in “Farmer-2”, whereas in “Moitree”, 72,588 genes have been assigned, with a total of 7060 FIG classes (Supplementary Table S1).

### 2.5. Comparison of the N Cycling Genes of Bacterial Communities in Lentil Cultivars: Moitree Vs. Farmer-2

**Figure 7:**
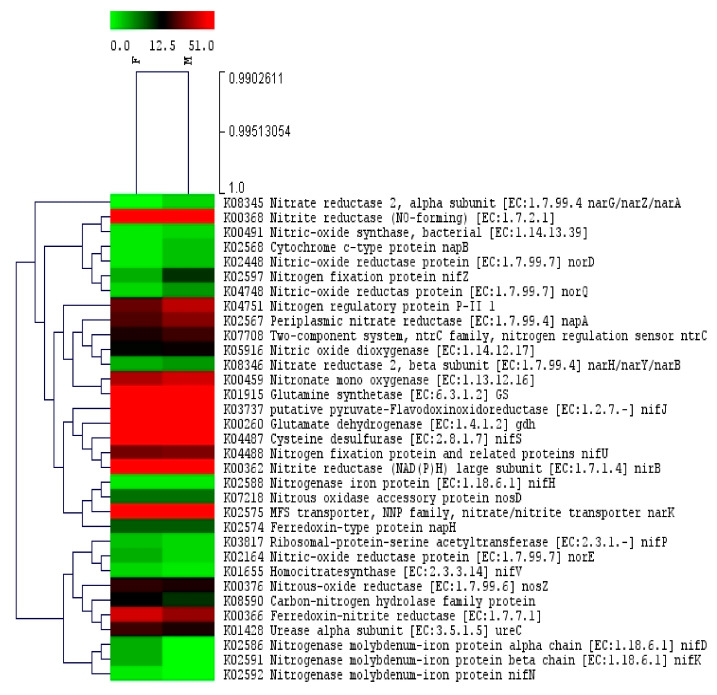
Heat tree of the 33 predicted genes of bacterial communities from lentil cultivars Moitree (M) and Farmer-2 (F) related with nitrogen cycling process.

The functional profiles of samples from “Farmer-2” and “Moitree” were further analyzed with special emphasis on genes predicted to be linked with N cycling based on KO group assignments (Supplementary Table S2) and are presented through a heat tree (Figure 7).

Among the gene families, glutamine synthetase *GS*, glutamate dehydrogenase *gdh*, cysteine desulfurase *nifS*, nitrite reductase (NO-forming), nitrite reductase (NAD(P)H) large subunit *nirB*, putative pyruvate-flavodoxin oxidoreductase *nifJ*, nitronate mono oxygenase *nmo*, MFS transporter, and NNP family nitrate/nitrite transporter *narK* were the most abundant gene families of the bacterial communities associated with both cultivars. The samples from “Moitree” had a relatively high abundance of genes related to denitrification processes (periplasmic nitrate reductase *napA*, nitrite reductase (NO-forming), cytochrome c-type protein *napB*, nitrate reductase 2 alpha subunit *narG*, and nitrate reductase 2 beta subunit *narH*). In both cultivars, low abundance was detected for nitric-oxide reductase protein (*norD*, *norE*, and *norQ* genes) responsible for denitrification of NO_2_^−^ to N_2_. Our results revealed higher abundance of nitronate mono oxygenase *nmo*, urease alpha subunit *ureC*, glutamate dehydrogenase *gdh*, and especially glutamine synthetase *GS* in “Moitree”, which are involved in metabolism of organic N. Genes related to BNF, such as nitrogenase iron protein *nifH*, *nifZ*, *nifU*, and related proteins; ribosomal-protein-serine acetyl transferase *nifP*; putative pyruvate-flavodoxin oxido reductase *nifJ*; homocitrate synthase *nifV*; cysteine desulfurase *nifS*; nitrogenase molybdenum-iron protein alpha chain *nifD*; nitrogenase molybdenum-iron protein beta chain *nifK*; and nitrogenase molybdenum-iron protein *nifN* were also identified. The *nifH* gene is required for functional nitrogenase in almost all diazotrophs, and it was observed in equal frequency in both cultivars. An abundance of *nifZ*, *nifJ*, and *nifS* genes was higher in “Moitree” as compared to “Farmer-2”, whereas the *nifD*, *nifK*, and *nifN* genes were present only in “Farmer-2”.

### 2.6. Comparison of the P Cycling Genes of Bacterial Communities in Lentil Cultivars: Moitree vs. Farmer-2

**Figure 8:**
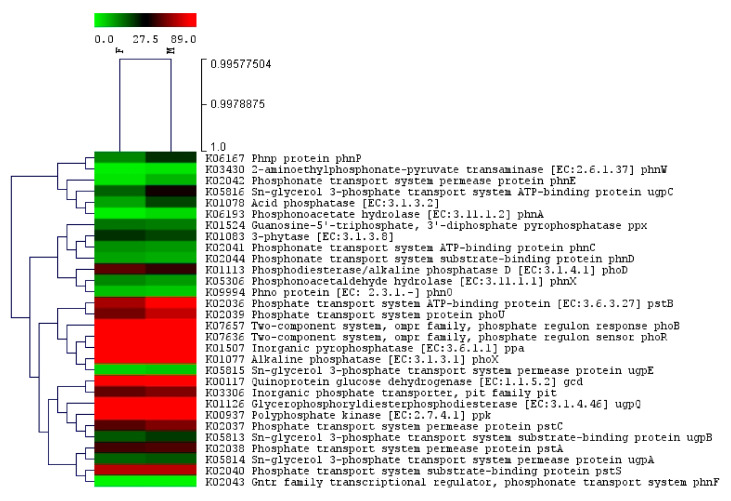
Heat tree of 30 genes of bacterial communities related to the phosphorus cycling process in lentil cultivars Moitree (M) and Farmer-2 (F).

The combined abundance of predicted genes involved in P cycling was quantified through KO group assignments among the samples collected from both the cultivars (Supplementary Table S3) and presented through a heat tree (Figure 8). The heat tree revealed significant differences in the composition of the P cycling bacterial communities and the composition of the P cycling functional genes between the cultivars (*p* = 0.0002). The abundance of almost all genes involved in phosphate esterase activity (alkaline phosphatase *phoX* and glycerophosphoryl diester phosphodiesterase *ugpQ*), phytase activity (two-component system, ompr family-related genes viz. *phoR*, *phoB*, and phosphate transport system protein *phoU*), phosphonate degradation (2-aminoethyl phosphonate-pyruvate *phnW*, phosphonoacetate hydrolase *phnA*, and phnp protein *phnP*), inorganic phosphate solubilization (quinoprotein glucose dehydrogenase *gcd*, polyphosphate kinase *ppk*, and inorganic pyrophosphatase *ppa*), and phosphorus transportation (phosphonate transport system permease protein *phnE*; Sn-glycerol 3-phosphate transport system proteins viz. *ugpA, ugpB, ugpC*, and *ugpE*; phosphate transport system ATP-binding protein *pstB*; phosphate transport system permease proteins *pstA* and *pstC*; and inorganic phosphate transporter, pit family protein) were more in the case of “Moitree” in comparison to “Farmer-2”, whereas phosphodiesterase/alkaline phosphatase *phoD***,** 3-phytase, guanosine-5′-triphosphate 3′-diphosphate pyrophosphatase *ppx*, phosphonoacetaldehyde hydrolase *phnX*, phosphonate transport system proteins viz. *phnC* and *phnD*, and Phno protein *phnO* showed lower abundance in “Moitree” as compared to “Farmer-2”.

## 3. Discussion

The relationship between plants and their rhizospheric microbiota is gaining importance for proper understanding of the mechanism underlying microbial involvement in boost plant growth as well as subsequent tailoring of microbiome composition aiming towards improvement in agricultural productivity. Plant roots secrete a plethora of exudates which vary with plant species [38], ecotypes [39], age of the plant [40], and soil type [41]. Unprecedented progress in the Next Generation Sequencing (NGS) platforms has made it feasible to get deeper insight into the abundance and diversity of rhizospheric niche, a hotspot of ecological richness [42] considering culturable (<1% of total population) and non-culturable microbes [43]. In our study, an attempt was made to characterize the microbial community profiles of two lentil cultivars that showed differential performance under rice-fallow ecology. Several host–genotype-specific rhizospheric microbial community developments have been studied in many crops viz. peanut [6], soybean [11], sweet potato [35], tomato [44], rice [45], mulberry [46], and maize [47]. Most of the studies revealed that plant genotype plays a very small but significant role in shaping rhizospheric microbial association under diverse agroecological situations [48,49] in order to mitigate different stress conditions like phosphate starvation [50], nutrient mobilization [51], and plant growth promotion [52]. Rice-fallow is a unique ecology in humid tropics and subtropics that account for a considerable cultivable land. To our knowledge, this study is the first attempt to investigate the host–genotype interaction on rhizosphere microbiome profiling of lentil cultivars in rice-fallow ecology using the whole metagenomic approach.

In the present study, the tested lentil cultivars, Moitree and Farmer-2, exhibited differential response regarding N and P content in the tissues as well as enzymatic activities in relation to BNF and P cycling. P is the major limiting factor in the soil as about 30–80% of the total P is present in organic forms [53]. Efficient acquisition and utilization of this group of P require dephosphorylation by various ubiquitous classes of enzymes viz. phytases and phosphatases before assimilation by the plants [54,55]. Higher P content in the grains of “Farmer-2” coupled with better phytase and phosphatase activities in plant and soil (Table 1 and Table 2) revealed that this cultivar could have developed better “P mining capacity” through enhancing solubilization and mineralization of P from sparingly available pools in rice-fallow ecology. Cultivar Farmer-2 was introduced from the Mediterranean region with calcareous soil characterized by P deficiency [56], which might be triggering this cultivar to adopt several physiological mechanisms for improving “P mining capacity” in P limiting condition. Most of the nitrogen fixing legumes have unique potential to change the rhizospheric pH through exudation of organic acids, and as there is also variation in a rhizosphere redox potential which can trigger P acquisition. In the present study, the impact of all these factors was not investigated, which warrant integration of these factors in future investigations. Variation was detected regarding phosphatase and phytase activity among lentil cultivars in the present study, which corroborates the findings in other legumes [57,58]. On the contrary, “Moitree” adopted improved “P acquisition efficiency” through developing a prolific root system and thus enabled better root foraging capacity and higher yield under rice-fallow ecology. Earlier studies carried out in lentil as well as in other food legumes suggested the prevalence of genotypic variation for root architecture including root length and the presence of dense root hairs, which further influence soil exploration for availability of moisture and less mobile nutrients like P [59,60,61]. The cultivar Moitree exhibited higher nutrient translocation efficiency along with higher N content in the grains (Table 1). Our data revealed that LB activity was higher in “Moitree” compared to “Farmer-2”. Corroborated by the present findings, previous studies also reported significant positive correlation between the LB concentration in legume nodules and total N in plant aerial part [62,63]. The allantoin and allantoic acid are the groups of ureides which are the major nitrogenous products of BNF in legumes and act as a determining factor regarding the BNF ability of the nodulated legume [64,65]. A higher concentration of LB and allantoin in “Moitree” thus reflected a better N fixing ability in comparison to “Farmer-2”.

In our study, Proteobacteria, Cyanobacteria, Bacteroidetes, Actinobacteria, and Acidobacteria were the most dominant bacterial phyla detected in rhizosphre of two lentil cultivars. Previous studies also reported a plethora of Proteobacteria, Acidobacteria, Bacteroidetes, and Actinobacteria in legumes like soybean and peanut [6,11]. Alphaproteobacteria were relatively more abundant in lentil cultivar “Farmer-2” previously identified as the dominant phylum in waterlogged rice soil [66]. Interestingly, *Paenibacillus* was found only in “Farmer-2”. This group is known as Plant Growth-Promoting Rhizobacteria (PGPR), which can solubilize phosphate [67]. Besides *Paenibacillus*, a number of other well-described phosphate solubilizing plant growth promoting bacteria (PGPB) including *Pseudomonas* and *Lysobacter* have been identified to improve various growth promoting activities including phosphate solubilization [68,69]. The results based on taxonomic abundance of the bacterial dominant group present among the cultivars could not reveal any meaningful conclusion regarding the congruous existence of specific rhizospheric bacteria. It is evident from this study that genotypes have a diminutive contribution towards shaping microbiome diversity [6]. On the contrary to that, previous studies reported a significant role of genotypes in selecting specific microbial groups in the rhizosphere [10,34,35]. Considering the functional profiling of a rhizosphere microbiome, our study has identified redundancy in the metabolic capacities of the bacterial communities with dominance of genes related to the metabolism and genetic processing categories. The present study is in accordance with the previous findings supporting the expression of microbial house keeping genes towards governing microbial transcriptional activity in soil [10,70]. The present finding warrants conducting a comprehensive study on functional annotation based on taxonomic classification towards drawing a conclusion regarding the specific function of a rhizospheric microbiome.

Diversification in the composition of root exudates among different plant species or cultivars within the same species plays a pivotal role in communication and further recognition that ultimately tailors distinct colonization of microbiota as per the need of the host and vice versa [71]. Lentil thrives basically on a nominal input-oriented system in rice-fallow, where rhizospheres’ microbiomes have to play a crucial role in increasing the bioavailability of native soil nutrients, especially N and P [72,73]. Any cultivar possessing the capacity to utilize native soil nutrients would perform better under this situation. The native nutrient utilization efficiency of lentil cultivars was determined through identification of genes in the microbiome responsible for the nutrient cycling process. In the present study genes, related to the N cycling process revealed no significant differences (*p* = 0.06) among the two tested cultivars though their nutrient foraging pattern varied owing to their differential root systems. Denitrifying microbial diversity (both classified and unclassified) as well as the abundance of denitrification process-related marker genes (*napA* and *narG*) were more in “Moitree” in comparison to “Farmer-2” (Figure 8 and Supplementary Table S2). Gene distribution related to the N cycle showed that the denitrification played a crucial role in “Moitree”. The higher expression of *GS* and *ureC* in “Moitree” confirmed that the assimilatory denitrification metabolism was highly upregulated in this cultivar in comparison to “Farmer-2”, which further validated/was supported through reduced N depletion as urease an enzyme involved in N mineralization [10]. Thus, “Moitree” attributed higher N content in grains (Table 1). Being a leguminous crop, genes related with BNF were expected and identified in both the cultivars which were in harmony with previous reports in soybean [11] and pea [74]. Most of the genes responsible for BNF were detected in almost equal frequencies in “Moitree” and “Farmer-2”, though *nifZ*, *nifJ*, and *nifS* genes, which are the minor operons of *nif* genes, were present in higher abundance in “Moitree”. The presence of the *nifD, nifK*, and *nifN* genes which encode component-I of Mo-nitrogenase complex (Fe-Mo) was detected only in “Farmer-2” and confirmed the differences in predicted abundance of genes related to BNF [75]. This finding is in accordance with previous reports indicating the role of plant genotypes in determining the microbiome composition with different BNF efficiencies or in regulating the microbial machinery involved in BNF [6,76].

In legumes, P is the major yield-determining nutrient. Rhizosphere-associated microbes impart a crucial role in mobilization of recalcitrant forms of P [77]. Functional annotation of the metagenomic datasets revealed the importance of the microbial P cycling process in the studied lentil cultivars with signifiant differences (*p =* 0.0002) in the P cycling functional genes of the microbiome. It can be assumed that “Moitree” with a profuse root system enables better nutrient foraging as well as improved efficiency to recruit more microbiota as compared to “Farmer-2”; the P solubilizing capacity of “Farmer-2” was greaeter, but microbial count was less. Limited information impedes our understanding towards the influence of heteroginities of root architecture on microbiome composition and diversity in the plant rhizosphere [77]. A recent study conducted in peach trees clearly revealed how different categories of root modulate the composition and count of rhizospheric microbial communities [78]. It was observed that phosphate esterase activity, phytase activity, phosphonate degradation, inorganic phosphate solubilization, and phosphorus transportation activity were more evident in “Moitree” than in “Farmer-2” (Figure 8 and Supplementary Table S3) and catalyze the hydrolysis and release of available P to plants [79]. Though the predicted gene expression related to *phoX* was higher in “Moitree”, in ‘’Farmer-2’′, the number of *phoD* transcripts was higher. The latter is a class of alkaline phosphatase which plays an important role in the release of plant-available inorganic P from organic P in soil [80]. Among the phosphoesterase class of enzymes, alkaline phosphatase and glycerol phosphoryl diester phosphodiesterase were in abundance in both cultivars, indicating that perhaps these enzymes have a higher tendency for P mineralization in rice-fallow ecology with neutral reaction as compared to acid phosphatase and phytase enzymes, as supported by the earlier finding [81]. In the present study, a large number of different subunits of inorganic phosphate solubilization process-related genes (*gcd*, *ppk*, and *ppa*) and P transporter-related genes (*Pst* and *Pit*) have been detected in higher abundance in “Moitree”, which empowered the soil microbial communities to efficiently utilize and internalize P into their metabolic processes so that the unavailable form of P is converted into available forms to the plant [82,83]. Therefore, a more detailed evaluation of the sequences related to P cycling revealed a predominance of genes linked to P metabolism, suggesting direct solubilization of P and, therefore, immediate availability to the crop.

## 4. Materials and Methods

### 4.1. Experimental Site and Soil Sampling

The present experiment was carried out at the Regional Research Sub-Station (RRSS) of Bidhan Chandra Krishi Viswavidyalaya located at Chakdah (Lat: 23°5.3′ N; Long: 83°5.3′ E and altitude of 9.75 m above msl), Nadia district, West Bengal, India during the winter season of 2018–2019, where a long-term rice-based food legume cropping system has continued for the last six years. The soil of the experimental plot is sandy clay loam having pH: 7.05, organic C: 6.8 g/kg available N: 154 kg/ha, available P: 16 kg/ha, and available potassium:129 kg/ha. Genetically, pure seeds of 130 lentil cultivars were sown under rice-fallow situation after the harvest of monsoon rice, leaving 20 cm standing stubbles in the field by opening a narrow furrow in between two rows of rice using a manual furrow opener. The crop was raised following agronomic practices with mineral fertilizers (fertilizer dose: N:P_2_O_5_:K_2_O = 20:40:40 kg/ha) for normal crop growth. Finally, two cultivars, namely “Moitree” (WBL-77) and “Farmer-2”, were selected based on their differential performance under rice-fallow ecology for further analyses. “Moitree” is a well-adapted variety in rice-fallow ecology of Gangetic delta evolved from a cross between ILL 7723 and BLX 88176 with small seed size (microsperma) and having 115–120 days duration. “Farmer-2” is a landrace of the Mediterranean region and a newly introduced line to Indian ecology by ICARDA (International Center for Agricultural Research in the Dry Areas) with seed size larger than “Moitree” and 130–135 days crop duration.

Three representative plants with complete root system from both cultivars were excavated manually using *augar* at the maximum vegetative stages (45 days after sowing/seeding) and cleaned thoroughly following the method described earlier [84]. After washing, shoots and roots were separated and dried in oven at 70 °C till constant weight was achieved. Triplicate soil samples were also collected randomly from the rhizospheres of both cultivars at the maximum vegetative stage. One part of the sample was air dried, sieved through a 2-mm sieve, and kept in a polythene bag for soil chemical analysis. The second part of the soil sample was kept in a refrigerator at 4 °C for soil enzyme analysis. The remaining third part of the soil sample was homogenized by mixing properly, after removal of roots and debris for DNA extraction. Finally, the root balls of lentils were removed very carefully and the soil loosely attached to the roots was removed by gentle shaking. Roots with tightly associated soil were put into a 50-mL centrifuge tube filled with 30-mL of autoclaved phosphate buffer pH. The tube was vortexed at maximum speed for 1 min, and the slurry was filtered through a 100-μm cell strainer into a new 50-mL centrifuge tube. The soil slurry was then centrifuged to precipitate soil particles. After another round of resuspension and centrifuging, the soil pellet was collected into 1.5-mL microcentrifuge tubes and snap chilled in liquid N for storage at −80 °C before DNA extraction.

### 4.2. Enzyme Assay of Soil Samples

Alkaline and acid phosphatase activities of the collected soil samples from both cultivars were estimated using standard protocol, as described by Tabatabai [85]. Soil phytase activity was estimated by measuring the amount of P_i_ released by hydrolysis using sodium phytate as the substrate [86].

### 4.3. Plant Analysis

Plant samples of both cultivars were first washed with running tap water followed by washing with double distilled water. Nodules were collected from each root sample of both cultivars for quantification. Roots and shoots were separated, and the fresh weight of each part was recorded. Further, shoot–root ratios were estimated by appraising the dry weight of shoot as well as root after drying inside a paper envelope in the oven at 70 °C until constant weight was recorded. The dried samples were ground into powder by using stainless steel grinder for further analysis. The total N content of the plant samples was analyzed by the Kjeldahl method using a Kel-Plus analyzer (Make: Pelican Equipments, Chennai, India), while the P content was analyzed through the Vanadomolybdate yellow color method [87]. Phytase activity was estimated colorimetrically by monitoring the release of P_i_ from phytic acid (Na-InsP6; Sigma-Aldrich, St. Louis, MO, USA), as per the standard protocol [82]. The acid phosphatase activity was determined following the protocol of Gilbert et al. [88]. The leghaemoglobin (LB) in fresh nodules was extracted from the root systems of both cultivars using sodium phosphate buffer (pH 7.4) and estimated following the standard protocol [89]. Finally, the content of allantoin was determined following the protocol of Vogels and Van Der Drift [90], in which ureides were extracted from both the root samples and were subjected to estimation of endogenous glyoxylate, allantoic acid-derived glyoxylate, and allantoin-derived glyoxylate. The concentration of allantoin was estimated by subtracting the amount of endogenous glyoxylate from the total amount of glyoxylate in each sample. The root trait parameter of both cultivars was measured using a scanner and the WINRHIZO software package (REGENT Instruments Inc., Quebec City, QC, Canada).

### 4.4. DNA Extraction, Library Preparation, and Metagenomic Sequencing

Soil DNA was isolated from both cultivars by Xcelgen Soil DNA isolation Kit. The quality of DNA was checked on a 0.8% agarose gel, and the concentrations were determined using Qubit^®^ 2.0 Fluorometer. The paired-end sequencing libraries were prepared using New England Biolabs^®^ NEB Next Ultra DNA Library Prep Kit (Catalog# E3730) for Illumina (San Diego, CA, USA). After obtaining the Qubit concentration for the library and the mean peak size from Bioanalyser profile, the library was loaded onto the Illumina platform for cluster generation and sequencing. Paired-end sequencing allowed the template fragments to be sequenced in both the forward and reverse directions. The average size of the libraries were 447 bp and 395 bp for “Farmer-2” and “Moitree”, respectively. The libraries were sequenced on Illumina NextSeq 500 sequencer (Illumina, San Diego, CA, USA, 2 × 150 bp chemistry) to generate about 4–5 GB data for each sample. Read count summaries and assembly statistics are provided in Table 4. All raw metagenomics datasets have been submitted to the NCBI Sequence Read Achieve database (BioProject Accession Number: PRJNA639655).

### 4.5. Metagenomic Sequence Assembly, Gene Prediction, Taxonomy, and Functional Annotation

Paired-end raw sequences were quality filtered to remove adapter sequences, following earlier literature [91]. The scaffolds generated for the two soil samples from both cultivars using Metaspades program of the SPADes assembler [92] were subjected to open reading frames (ORFs) prediction using Prodigal (v2.6.3) [93]. The final ORFs obtained from Prodigal were used further for assigning taxonomic ranking combining the lowest common ancestor (LCA) algorithm in Kaiju (http://kaiju.binf.ku.dk/server) [94], where reads were compared to the nonredundant protein database “nr” used by NCBI BLAST with maximum *e*-value of 1 × 10^−5^, run mode-Greedy, minimum alignment length of 11, and maximum alignment length of 75 and allowed mismatches of up to 5. For identification of read belonging to 16 S sequences, Parallel-meta tool was executed on FASTQ files for both samples followed by mapping on the SILVA database. The FASTA files for 16 S sequences were converted into FASTQ files using inhouse-scripts for use as input for QIIME (Quantitative Insight Into Microbial Ecology) analysis to get taxonomic summaries integrating Python and R scripts and Qiime v.1.9.1 [95]. The Krona tool was used for plotting graph (Supplementary Figures S3 and S4) for each cluster [96]. All sequences from the two samples have been clustered into OTUs based on their 97% sequence similarity using UCLUST algorithm [97]. For comparison of the functional profiles for the microbial metagenomes of “Moitree” and “Farmer-2”, COGNIZER (v0.9b) [98] was used at default parameters which simultaneously provide COG, KEGG, Pfam, GO, and FIGfams annotations to individual sequences constituting metagenomic datasets. Genes related to N and P metabolism from each sample were selected within KO using a maximum e-value of 1 × 10^−5^ having a minimum identity of 80%.

### 4.6. Statistical Analysis

Experimental results regarding plant and soil samples were expressed as mean ± standard error of mean considering three replicates for all measurements. Independent sample “t-test” was carried out to analyse individual plant traits, enzyme activity, as well as number of predicted microbiome genes related to N and P cycling processes. All statistical analyses were performed in R version 3.5.0 [99]. Alpha diversity was calculated considering the OTU richness for getting Shannon index, observed species, and Chao 1 estimate among the bacterial communities associated with the two cultivars. The rank abundance curve was generated for representing species richness and species evenness among the cultivars. A rarefaction curve was portrayed to depict the species richness of the samples. A PCA plot was generated at reads and abundance level for phylum in both samples using the variance-covariance matrix model. Further, the N number of bootstrap simulations where N = 1000 was deployed for increasing accuracy of PCA plots. Heat trees were generated for the N- and P-related genes among the samples using MeV4.8.1 tool and the R package pheatmap (version 1.0.10).

## 5. Conclusions

Our results revealed that lentil cultivars, Moitree and Farmer-2, exhibited trivial differences in diversity and function of microbiomes related to N metabolism in their rhizosphere. A higher abundance of genes associated with denitrification process was detected in “Moitree”; however, genes related to BNF exhibited almost equal distribution in both cultivars. Contrarily, the microbiome P cycling genes exhibited significant differences among the cultivars. Thus, it can be concluded that the availability of P is the major determining factor towards better performance of the genotype under rice-fallow ecology. Both the tested cultivars exhibited differential mechanisms to use the native P under rice-fallow ecology. The cultivar Moitree with profuse root system and high shoot–root biomass exhibited better “root foraging capacity” towards improved acquisition of native P coupled with better N turnover under rice-fallow ecology. Most of the rice-fallow is deficient in available P due to high moisture content in soil. Hence, “Moitree” could perform better under nutrient poor soil in rice-fallow ecology. On the contrary, the newly introduced “Farmer-2” from the Mediterranean region into the Indian subcontinent exhibited greater P content in roots as well as in grains along with good phosphatase and phytase activity in both plant and soil. This could shed some lights on the fact that the genetic makeup of this cultivar enabled better “P mining capacity” for solubilization of the fixed P into plant available form followed by efficient transportation of P from source to sink though its availability at the root/soil interface was poor due to retarded expression of the microbiome-associated P cycling genes in the rhizosphere. Our efforts for a single factor explanation regarding cultivar specific microbiome recruitment followed by nutrient availability exerted limited impact towards explaining the complex natural system. Comprehensive research is further needed regarding nutrient interaction integrating the role of the rhizosphere-associated microbiome for a better understanding of nutrient availability under rice-fallow ecology.

## Figures and Tables

**Table 1:** Plant attributes studied in two lentil cultivars under rice-fallow ecology.

Plant Attributes	Moitree Mean ± SE	Farmer-2 Mean ± SE	*p* Value
GNC	4.47 ± 0.23	3.64 ± 0.19	0.001
GPC	2.61 ± 0.13	2.74 ± 0.14	0.223
RNC	0.65 ± 0.03	0.98 ± 0.05	<0.0001
RPC	0.96 ± 0.05	1.72 ± 0.09	<0.0001
SRR	8.41 ± 0.58	4.63 ± 0.24	<0.0001
ACP	7.85 ± 0.40	9.78 ± 0.50	0.001
PP	3.90 ± 0.20	6.30 ± 0.32	<0.0001
LB	0.22 ± 0.0011	0.14 ± 0.0007	<0.0001
Allantoin	50.36 ± 2.57	43.93 ± 2.24	0.009
NNP	33 ± 1.68	24 ± 1.22	<0.0001
TRL	38.97 ± 2.42	22.17 ± 1.65	<0.0001
RSA	29.7 ± 1.51	14.6 ± 0.74	<0.0001
RD	0.7 ± 0.04	0.5 ± 0.03	<0.0001

SE: standard error of mean, GNC: grain nitrogen concentration (%), GPC: grain phosphorus concentration (%), RNC: root nitrogen concentration (%), RPC: root phosphorus concentration (%), SRR: shoot–root ratio (wt/wt), ACP: acid phosphatase (µ mol/hr/g), PP: phytase (%) LB: leghaemoglobin (mg/g fresh weight), Allantoin: Allantoin content (mg/L), NNP: nodule number per plant, TRL: average tap root length (cm), RSA: average root surface area (cm^2^), RD: average root diameter (mm).

**Table 2:** Enzymatic activity of rhizospheric soil between two lentil cultivars under rice-fallow ecology.

Genotype	ACP Mean ± SE	ALP Mean ± SE	PP Mean ± SE
Moitree	31.9 ± 1.63	27.8 ± 1.42	2.14 ± 0.11
Farmer-2	42.9 ± 2.19	36.9 ± 1.88	2.32 ± 0.12
*p* value	<0.0001	<0.0001	0.065

SE: standard error of mean, ACP: acid phosphatase (μg *p*-nitrophenol/g soil/ hr), ALP: alkaline phosphatase (μg *p*-nitrophenol/g soil/hr), PP: phytase (mg P/g soil/hr).

**Table 3:** Bacterial alpha diversity estimated by Shannon index, observed species, and Chao1 estimates using taxon resolution of 97% sequence similarity between lentil cultivars.

	Shannon Index	Observed Species	Chao1
Moitree	11.78	3947	4179.83
Farmer-2	11.39	3012	3467.01

**Table 4:** Read count summaries, assembly statistics, and gene prediction statistics between two lentil cultivars.

Assembly Elements	Moitree	Farmer-2
Total reads	58,363,086	32,085,250
Total bases	8,754,462,900	4,812,787,500
GC %	66.74	63.5
Total data (GB)	8.75	4.81
Scaffolds	105,291	121,422
Total scaffold length (bp)	125,203,059	93,836,361
Average scaffold length (bp)	1189.114	772.811
Scaffold N50 (bp)	1128	731
Max scaffold size (bp)	55,132	11,580
Genes	206,155	186,216
Total genes size (bp)	112,980,996	82,803,213
Average genes size (bp)	548.039	444.662
Max scaffold size (bp)	6315	4527

## Supplementary Materials

The following are available online at https://www.mdpi.com/1422-0067/21/23/8895/s1, Figure S1: Relative abundance of top 50 common bacterial taxa within microbial communities identified from lentil cultivars Moitree (M) and Farmer-2 (F) using R package P heatmap, Figure S2: Principal component analysis (PCA) based on shared common taxa profiles at phylum reads variance from lentil cultivars Moitree (M) and Farmer-2 (F), Figure S3: The Krona graph showing the relative abundance of annotated taxa in lentil cultivar Moitree, Figure S4: The Krona graph showing the relative abundance of annotated taxa in lentil cultivar Farmer-2, Table S1: Summery table representing comparative analysis between the samples of two lentil cultivars, Table S2: Summery of predicted genes from two lentil cultivars related with nitrogen (N) cycling under rice-fallow ecology along with t-Test: Paired Two Sample for Means, Table S3: Summery of predicted genes from two lentil cultivars related with phosphorus (P) cycling under rice-fallow ecology along with t-Test: Paired Two Sample for Means.

## Abbreviations

ACP	Acid phosphatase
ALP	Alkaline phosphatase
BLAST	Basic Local Alignment Search Tool
BNF	Biological nitrogen fixation
Bp	Base Pairs
C	Carbon
COG	Clusters of Orthologous Groups of proteins
FIGfams	Fellowship for the Interpretation of Genomes protein families
GB	Gigabyte
Gdh	Glutamate dehydrogenase
GLDC	Grain legumes & Dryland Cereals
GNC	Grain nitrogen concentration
GO	Gene Ontology
GPC	Grain phosphorus concentration
GS	Glutamine synthetase
ICAR	Indian Council of Agricultural Research
ICARDA	International Centre for Agricultural Research in the Dry Areas
KAAS	KEGG Automatic Annotation Server
KEGG	Kyoto Encyclopedia of Genes and Genomes
KO	KEGG Orthology
LB	Leghaemoglobin
LCA	Lowest common ancestor
N	Nitrogen
NCBI	National Center for Biotechnology Information
NGS	Next Generation Sequencing
nif	Nitrogen fixation
nmo	Nitronate mono oxygenase
NNP	Nodule number per plant
nor	Nitric-oxide reductase
Nr	Non-redundant
ORFs	Open reading frames
OTUs	Operational taxonomic units
P	Phosphorus
PCA	Principal Component Analysis
Pfam	Protein Families
PGPB	Plant growth promoting bacteria
PGPR	Plant Growth-Promoting Rhizobacteria
PP	Phytase
QIIME	Quantitative Insight Into Microbial Ecology
RD	Average root diameter
RNC	Root nitrogen concentration
RPC	Root phosphorus concentration
RSA	Average root surface area
SE	Standard error of mean
SRR	Shoot–root ratio
