# Supplementary material for: Metagenomic Insights into Rhizospheric Microbiome Profiling in Lentil Cultivars Unveils Differential Microbial Nitrogen and Phosphorus Metabolism under Rice-Fallow Ecology
Source: Int J Mol Sci. 2020 Nov 24;21(23):8895. doi: 10.3390/ijms21238895 (PMC7727700; doi:10.3390/ijms21238895)

Supplementary Table S1: Summery table representing comparative analysis between the samples of two lentil cultivars

**Data**	**Moitree**	**Farmer-2**
No. of HQ reads(R1+R2)	58,363,086	32,085,250
No. of scaffolds	105,291	121,422
No. of Genes	206,155	186,216
Most abundant Phylum(# of Genes)	Actinobacteria (70,292)	Actinobacteria (54,137)
Most abundant Class(# of Genes)	Actinobacteria (49,547)	Actinobacteria (43,013)
Most abundant Order (# of Genes)	Propionibacteriales (19,836)	Propionibacteriales (18,509)
Most abundant Family(# of Genes)	Nocardioidaceae (19,495)	Nocardioidaceae (18,226)
Most abundant Genus (# of Genes)	*Nocardioides* (14,516)	*Nocardioides* (13,943)
Most abundant Species(# of Genes)	*Nocardioides iriomotensis* (5,993)	*Nocardioides iriomotensis* (5,601)
Gene Ontology (GO)(# of Genes)	165,424	147,839
Kyoto Encyclopedia of Genes and Genomes (KEGG)	140,995	120,946
Protein families (Pfam)	124,850	109,486
Clusters of Orthologous Groups of proteins (COG)	116,357	96,574
Fellowship for the Interpretation of Genomes (FIG)	72,588	61,058

Supplementary Table S2: Summery of predicted genes from two lentil cultivars related with N cycling under rice-fallow ecology along with t-Test: Paired Two Sample for Means

KO ids	Enzyme	Gene	No of genes in Moitree	No of genes in Farmer-2
K02588	Nitrogenase iron protein [EC:1.18.6.1]	*nifH*	1	1
K02567	Periplasmic nitrate reductase [EC:1.7.99.4]	*napA*	30	23
K00368	Nitrite reductase (NO-forming) [EC:1.7.2.1]	-	93	51
K02568	Cytochrome c-type protein	*napB*	3	1
K00376	Nitrous-oxide reductase [EC:1.7.99.6]	*nosZ*	17	19
K00459	Nitronate mono oxygenase [EC:1.13.12.16]	*nmo*	40	35
K02597	Nitrogen fixation protein	*nifZ*	10	4
K00366	Ferredoxin-nitrite reductase [EC:1.7.7.1]	-	32	40
K04751	Nitrogen regulatory protein P-II 1	-	37	26
K07218	Nitrous oxidase accessory protein	*nos*D	7	7
K07708	Two-component system, *ntrC* family, nitrogen regulation sensor	*ntrC*	21	17
K08590	Carbon-nitrogen hydrolase family protein	-	10	13
K00491	Nitric-oxide synthase, bacterial [EC:1.14.13.39	-	2	1
K02164	Nitric-oxide reductase protein [EC:1.7.99.7]	*norE*	2	4
K02448	Nitric-oxide reductase protein [EC:1.7.99.7]	*norD*	3	1
K04748	Nitric-oxide reductas protein [EC:1.7.99.7]	*norQ*	5	2
K05916	Nitric oxide dioxygenase [EC:1.14.12.17]	-	15	12
K02575	MFS transporter, NNP family, nitrate/nitrite transporter	*narK*	51	51
K08345	Nitrate reductase 2, alpha subunit [EC:1.7.99.4	*narG*/ *narZ*/ *narA*	2	0
K08346	Nitrate reductase 2, beta subunit [EC:1.7.99.4]	*narH*/ *narY*/ *narB*	5	4
K04488	Nitrogen fixation protein and related proteins	*nifU*	29	28
K03817	Ribosomal-protein-serine	*nifP*	2	3

	acetyltransferase [EC:2.3.1.-]			
K03737	putative pyruvate-Flavodoxin oxidoreductase [EC:1.2.7.-]	*nifJ*	58	53
K01655	Homocitrate synthase [EC:2.3.3.14]	*nifV*	1	2
K04487	Cysteine desulfurase [EC:2.8.1.7]	*nifS*	63	61
K01915	Glutamine synthetase [EC:6.3.1.2]	*GS*	241	203
K00260	Glutamate dehydrogenase [EC:1.4.1.2]	*gdh*	119	112
K01428	Urease alpha subunit [EC:3.5.1.5]	*ureC*	17	21
K00362	Nitrite reductase (NAD(P)H) large subunit [EC:1.7.1.4]	*nirB*	59	57
K02574	Ferredoxin-type protein	*napH*	8	8
K02586	Nitrogenase molybdenum-iron protein alpha chain [EC:1.18.6.1]	*nifD*	0	4
K02591	Nitrogenase molybdenum-iron protein beta chain [EC:1.18.6.1]	*nifK*	0	4
K02592	Nitrogenase molybdenum-iron protein	*nifN*	0	1
Mean			29.79	26.33
Variance			2262.55	1633.85
Observations			33	33
Pearson Correlation			0.99	
df			32	
t Stat			1.95	
**P(T<=t) two-tail**			**0.06**	
t Critical two-tail			2.04	

Supplementary Table S3: Summery of predicted genes from two lentil cultivars related with Phosphorus cycling under rice-fallow ecology along with t-Test: Paired Two Sample for Means

KO ids	Enzyme	Gene	No of genes in Moitree	No of genes in Farmer-2
K01077	Alkaline phosphatase [EC:3.1.3.1]	*phoX*	152	127
K01126	Glycerophosphoryl diester phosphodiesterase [EC:3.1.4.46]	*ugpQ*	120	105
K07636	Two-component system, ompr family, phosphate regulon sensor	*phoR*	128	105
K00937	Polyphosphate kinase [EC:2.7.4.1]	*ppk*	117	101
K01507	Inorganic pyrophosphatase [EC:3.6.1.1]	*ppa*	110	91
K07657	Two-component system, ompr family, phosphate regulon response	*phoB*	102	82
K00117	Quinoprotein glucose dehydrogenase [EC:1.1.5.2]	*gcd*	89	80
K02040	Phosphate transport system substrate-binding protein	*pstS*	66	66
K02036	Phosphate transport system ATP-binding protein [EC:3.6.3.27]	*pstB*	82	62
K02039	Phosphate transport system protein	*phoU*	68	52
K03306	Inorganic phosphate transporter, pit family	*pit*	54	48
K01113	Phosphodiesterase/alkaline phosphatase D [EC:3.1.4.1]	*phoD*	39	47
K02037	Phosphate transport system permease protein	*pstC*	54	46
K02038	Phosphate transport system permease protein	*pstA*	44	42
K01083	3-phytase [EC:3.1.3.8]	--	20	22
K05813	Sn-glycerol 3-phosphate transport system substrate-binding protein	*ugpB*	21	18
K05814	Sn-glycerol 3-phosphate transport system permease protein	*ugpA*	18	17
K05816	Sn-glycerol 3-phosphate transport system ATP-binding protein	*ugpC*	33	17
K01524	Guanosine-5'-triphosphate, 3'-diphosphate pyrophosphatase	*ppx*	14	15
K06167	Phnp protein	*phnP*	22	13
K05306	Phosphonoacetaldehyde hydrolase [EC:3.11.1.1]	*phnX*	11	13
K02041	Phosphonate transport system ATP-binding protein	*phnC*	11	12
K01078	Acid phosphatase [EC:3.1.3.2]	--	20	10
K02044	Phosphonate transport system substrate-binding protein	*phnD*	9	10
K09994	Phno protein [EC: 2.3.1.-]	*phnO*	6	7
K05815	Sn-glycerol 3-phosphate transport system permease protein	*ugpE*	6	5
K02042	Phosphonate transport system permease protein	*phnE*	8	3
K03430	2-aminoethyl phosphonate-pyruvate	*phnW*	3	2

	transaminase [EC:2.6.1.37]			
K06193	Phosphonoacetate hydrolase [EC:3.11.1.2]	*phnA*	4	2
K02043	Gntr family transcriptional regulator, phosphonate transport system	*phnF*	1	1
Mean			47.73	40.70
Variance			2015.86	1440.42
Observations			47.73	40.70
Pearson Correlation			0.99	
df			2	
t Stat			4.32	
**P(T<=t) two-tail**			**0.0002**	
t Critical two-tail			2.05	

Supplementary Figure S1: Relative abundance of top 50 common bacterial taxa within microbial communities identified from lentil cultivars Moitree (M) and Farmer-2 (F) using R package Pheatmap.

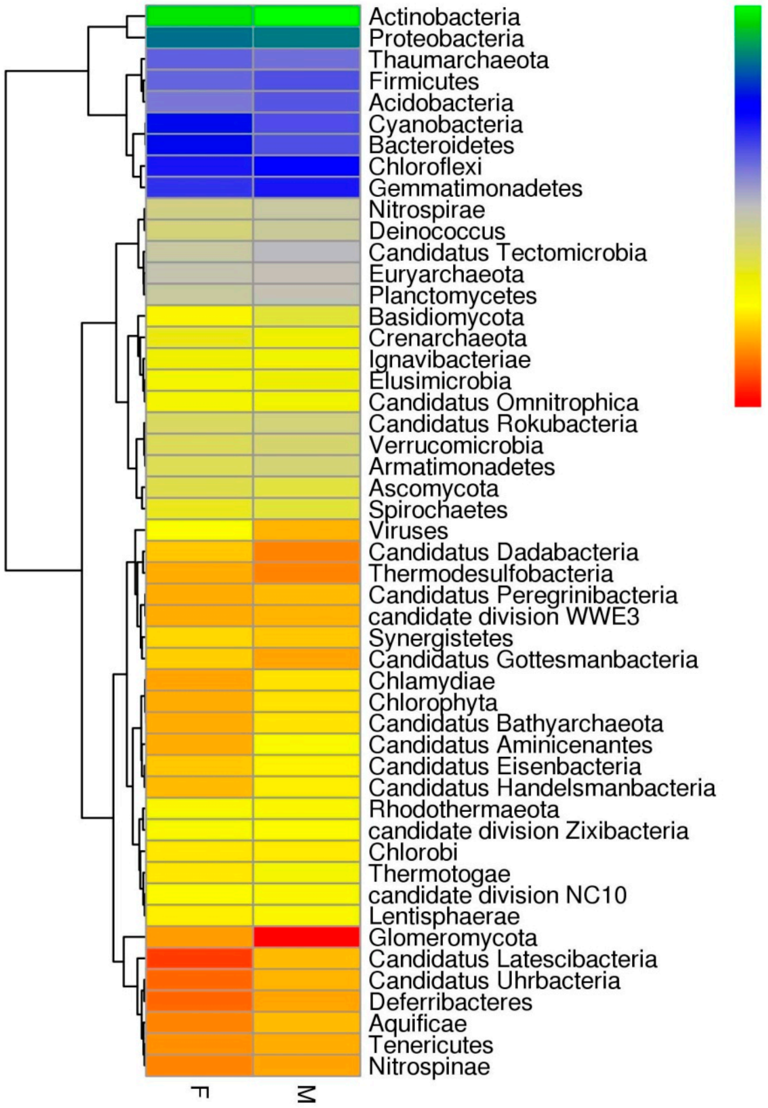

Supplementary Figure S2: Principal component analysis (PCA) based on shared common taxa profiles at phylum reads variance from lentil cultivars Moitree (M) and Farmer-2 (F).

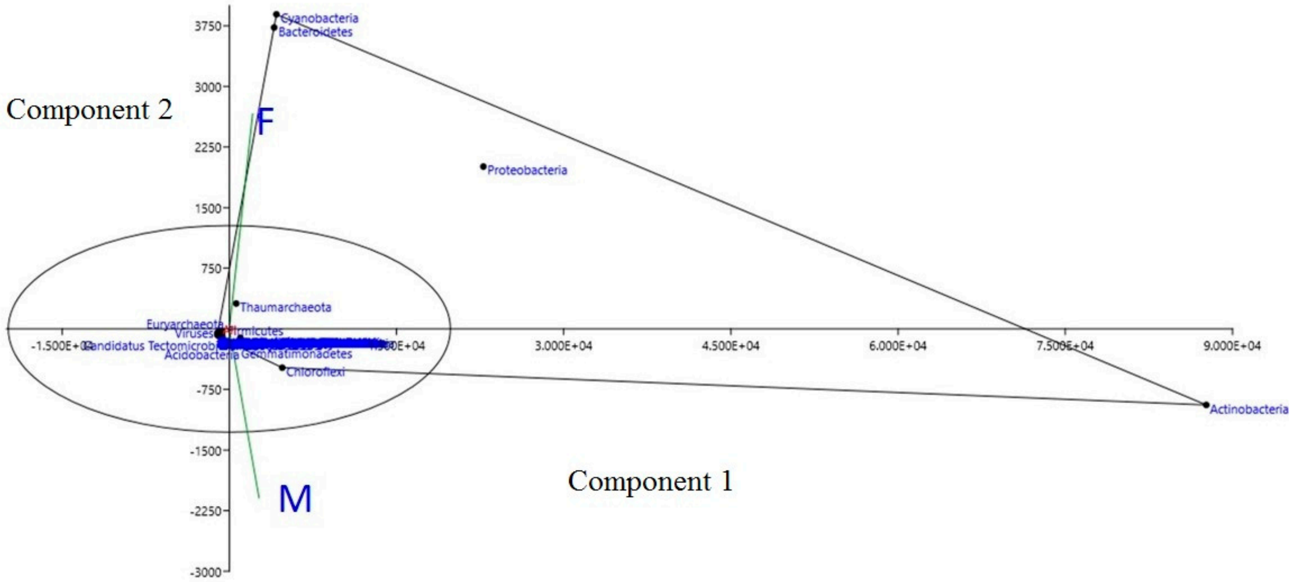

Supplementary Figure S3: The Krona graph showing the relative abundance of annotated taxa in lentil cultivar Moitree.

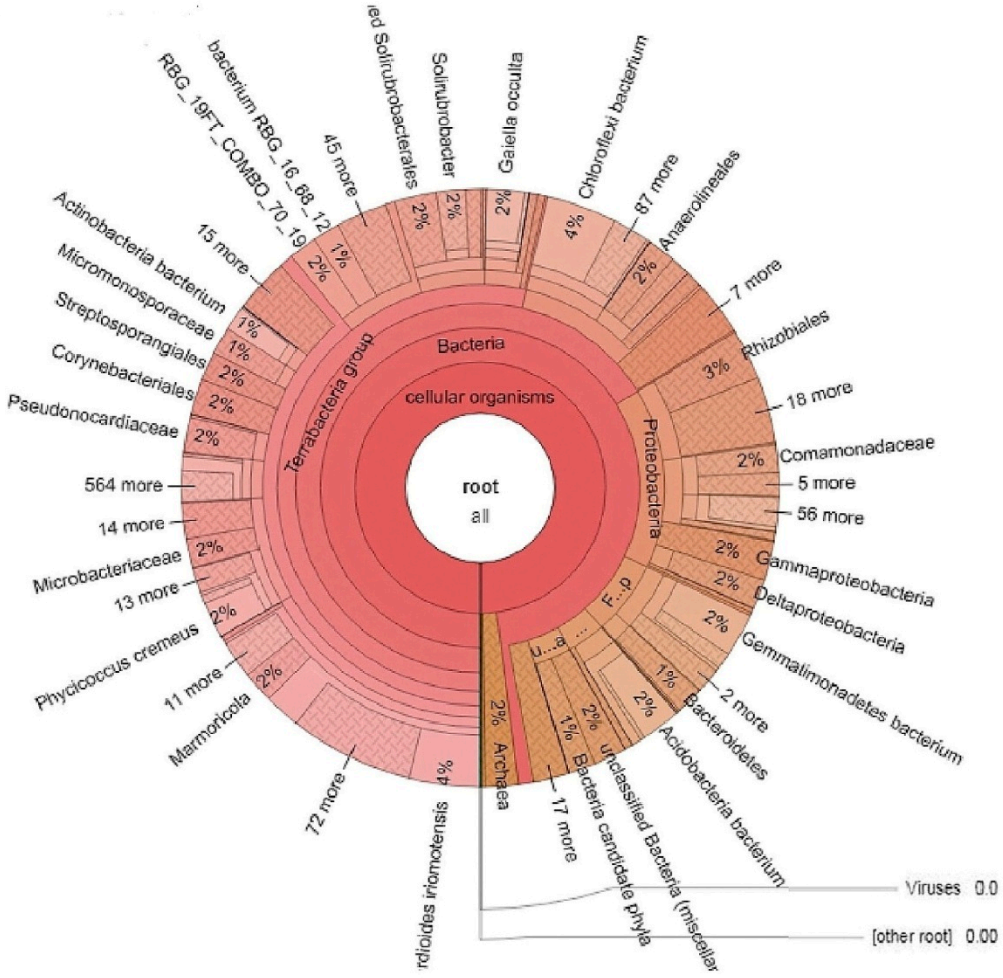

Supplementary Figure S4: The Krona graph showing the relative abundance of annotated taxa in lentil cultivar Farmer-2.

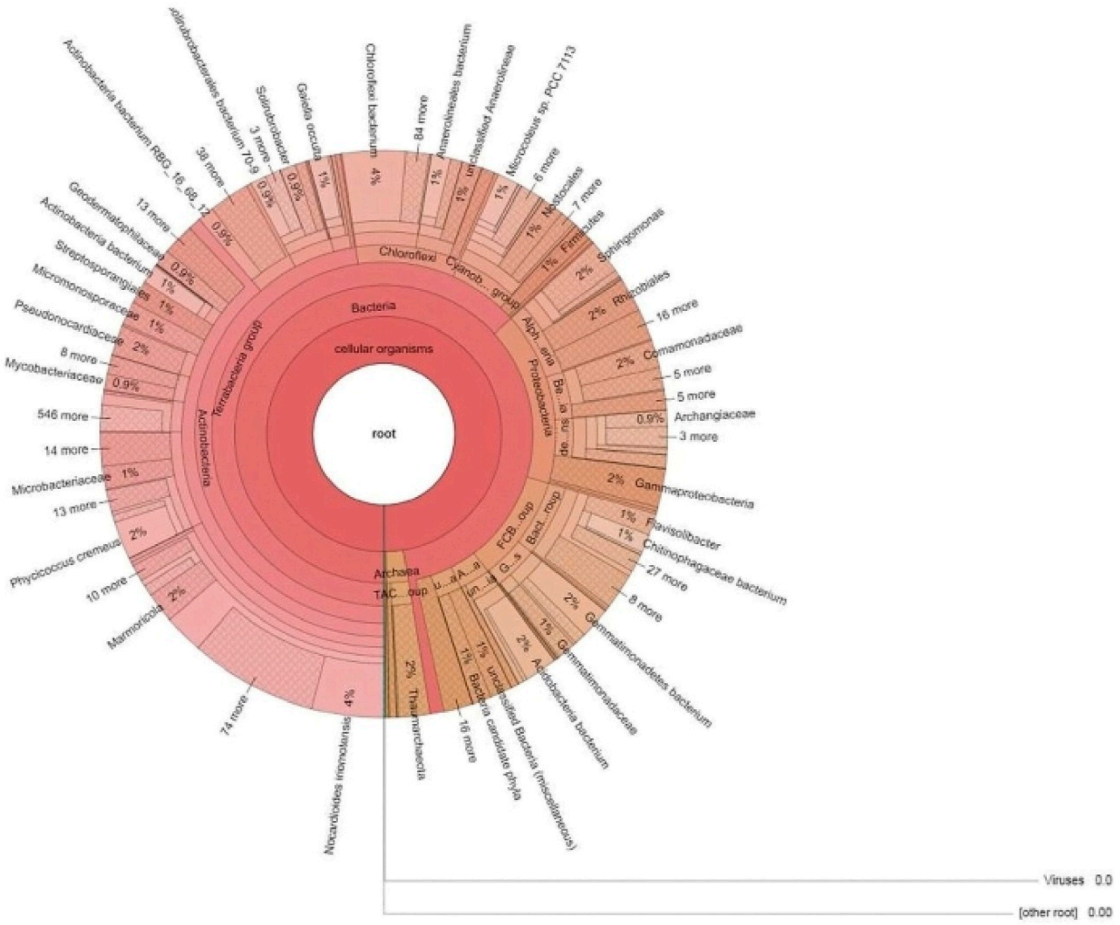

Supplement: Supplementary file 1 [file ijms-21-08895-s001.pdf]
